# Supplementary material for: Comparative multiomics analysis of cell physiological state after culture in a basket bioreactor
Source: Sci Rep. 2022 Nov 23;12:20161. doi: 10.1038/s41598-022-24687-4 (PMC9686226; doi:10.1038/s41598-022-24687-4)

2:27477448:-@NW_023666033.1:27476898:27477030:-@NW_023666033.1:27475687:27475876:-@NW_02366603

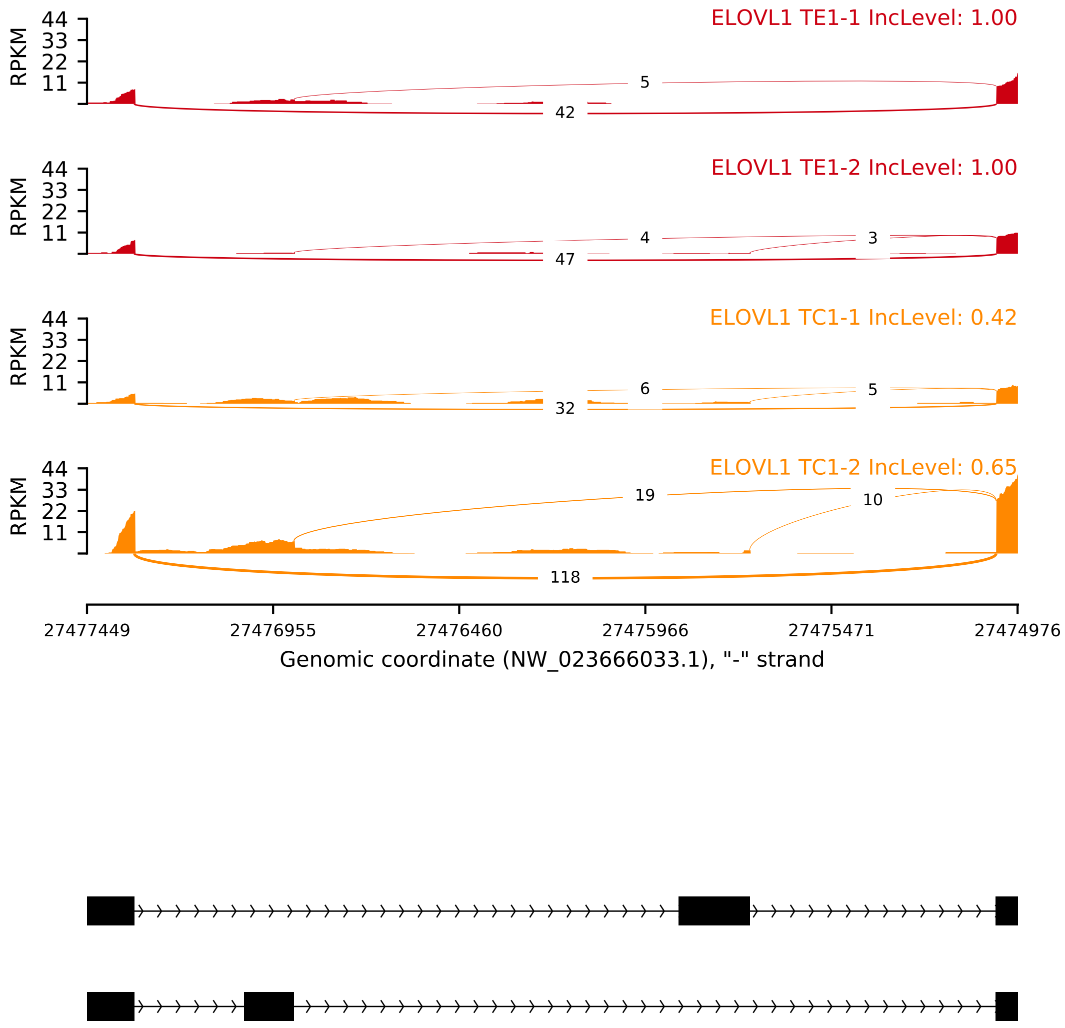

Supplement: Supplementary file 1 — Supplementary Information 1. [file 41598_2022_24687_MOESM1_ESM.zip › raw data/Transcriptomic raw data/7.AS/2.dasplot/TE1vsTC1/MXE/8_ELOVL1_NW_023666033.1_27477322_27477448_-@NW_023666033.1_27476898_27477030_-@NW_023666033.1_27475687_27475876_-@NW_023666033.1_27474974_27475033_-.pdf]
